# Hemosuccus Pancreaticus: A Rare Cause of Upper Gastrointestinal Bleeding in a Child With Recurrent Acute Pancreatitis and Splenic Artery Aneurysm

**DOI:** 10.7759/cureus.80863

**Published:** 2025-03-20

**Authors:** Fatima I Hsayan, Mohamad Al Ayoubi, Amani El Abed, Jennifer Abi Younes, Antoine S Geagea

**Affiliations:** 1 Gastroenterology, Lebanese University Faculty of Medicine, Beirut, LBN; 2 Internal Medicine, Lebanese University Faculty of Medicine, Beirut, LBN; 3 Pediatrics, Lebanese University Faculty of Medicine, Beirut, LBN; 4 Gastroenterology and Hepatology, Lebanese Hospital Geitaoui University Medical Center, Beirut, LBN

**Keywords:** gastro, hemosuccus pancreatic, pancreatitis, pseudoaneurysm of splenic artery, t cell acute lymphocytic leukemia

## Abstract

Hemosuccus pancreaticus is a rare cause of upper gastrointestinal bleeding, most commonly occurring in patients with acute or chronic pancreatitis. Other etiologies, such as pancreatic pseudocysts, pancreatic tumors, or iatrogenic injury from endoscopic ultrasound-guided fine needle aspiration (EUS-FNA), can also lead to wirsungorrhagia (bleeding from the ampulla of Vater), as described in hemosuccus pancreaticus.

We present the case of a child with acute lymphocytic leukemia who developed recurrent acute pancreatitis due to repeated treatment with Oncaspar, complicated by a pseudocyst and splenic artery aneurysm. His hospital course was further complicated by intermittent, massive upper gastrointestinal bleeding due to hemosuccus pancreaticus.

This case raises awareness and suspicion of hemosuccus pancreaticus, particularly in patients with acute or chronic pancreatitis. It also emphasizes the need for thorough investigation, given the rarity and diagnostic challenges of this condition. Additionally, we describe appropriate management through angiography and embolization of the splenic artery aneurysm, which successfully controlled the bleeding source.

## Introduction

Hemosuccus pancreaticus, defined as bleeding from the ampulla of Vater into the second portion of the duodenum via the pancreatic duct, is a rare condition [1]. The hemorrhage is usually intermittent and can range from an occult gastrointestinal bleed to a life-threatening hemorrhage [2,3]. It accounts for less than 1% of all upper gastrointestinal bleeds [4].

Melena is a common symptom, and patients may also develop abdominal pain, worsening anemia, or hemoptysis [2]. Approximately 80% of cases of hemosuccus pancreaticus result from pancreatitis (acute or chronic). Other etiologies include pancreatic tumors, pancreas divisum, pancreatic pseudocysts, and iatrogenic lesions of the pancreas. The splenic artery is the most commonly involved artery (approximately 60%) [1,4].

The diagnostic and therapeutic approach is challenging due to the anatomic origin of this pathology, intermittent upper gastrointestinal bleeding, and nondiagnostic computed tomography scans and endoscopies prior to angiography [1,2,4]. Computed tomography angiography is considered the gold standard for detecting the bleeding site, while angiography is the ideal diagnostic and therapeutic intervention [1,4].

Here, we report a rare case of hemosuccus pancreaticus in a child with complicated recurrent acute pancreatitis who developed intermittent, massive upper gastrointestinal bleeding.

## Case presentation

A 15-year-old boy was diagnosed 10 months ago with T-cell acute lymphoblastic leukemia. He was initially treated with cytarabine and methotrexate, followed by vincristine, doxorubicin, and Oncaspar (pegaspargase), completing five doses over a two-month period. The patient developed recurrent acute pancreatitis due to repeated Oncaspar doses, complicated by peripancreatic fluid collection, abdominal ascites, and diffuse necrosis and atrophy of the body and tail of the pancreas.

One month later, the patient presented to the emergency department with lethargy, abdominal distension, pain, and lower limb edema. Laboratory tests revealed severe anemia (hemoglobin (Hb) 7.5 g/dL, ref: 14-18 g/dL) and thrombocytopenia (platelets 27,000/mm³, ref: 130,000-400,000/mm³). He was admitted for further investigation and management. An urgent CT scan of the abdomen and pelvis with IV contrast was performed, revealing no evidence of active intra-abdominal bleeding within the previously described walled-off collection. Blood and platelet transfusions were initiated, which increased the Hb to 12.9 g/dL (ref: 14-18 g/dL) and platelets to 97,000/mm³ (ref: 130,000-400,000/mm³).

During hospitalization, the patient complained of dyspnea and an increase in abdominal girth. An urgent scan without IV contrast was performed, which demonstrated large abdominal ascites, an interval increase in the peripancreatic collection, and findings suggestive of hemoperitoneum. Active bleeding was ruled out with a normal CT scan of the abdomen and pelvis with IV contrast, as well as a normal arteriography. His hospital course was complicated by disseminated intravascular coagulation and respiratory distress, leading to his transfer to the intensive care unit, where he was intubated.

During his intensive care unit stay, the patient developed melena and a drop in Hb to 6 g/dL. Both esophagogastroduodenoscopy and colonoscopy were performed, revealing no signs of active upper gastrointestinal or colonic bleeding. The patient was stabilized after receiving appropriate blood transfusions. Ascites was drained percutaneously under CT guidance, and the drained fluid showed a high amylase level (4606 U/L), suggestive of pancreatic ascites. The patient was subsequently extubated and transferred to the regular floor.

**Figure 1:**
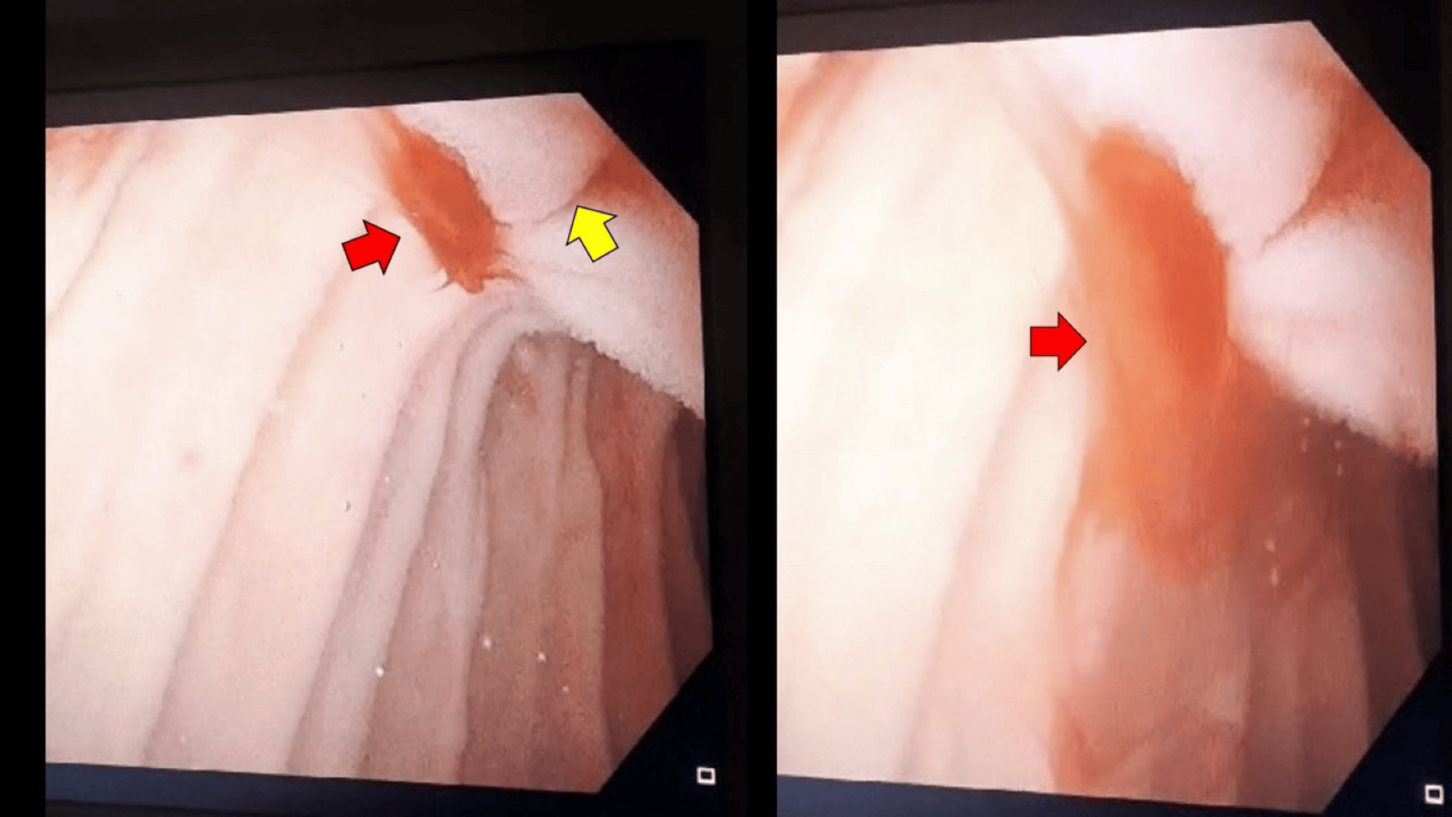
This figure, taken during gastroduodenoscopy, demonstrates active bleeding (red arrows) from the ampulla of Vater (yellow arrow) in the second part of the duodenum.

However, on the 29^th^ day of admission, he developed hematemesis. The gastroenterology team was consulted, and an urgent esophagogastroduodenoscopy was performed, which revealed massive fresh blood from the ampulla of Vater. As a result, hemosuccus pancreaticus was diagnosed, and the patient was referred for urgent angiography with embolization (Figure 1).

**Figure 2:**
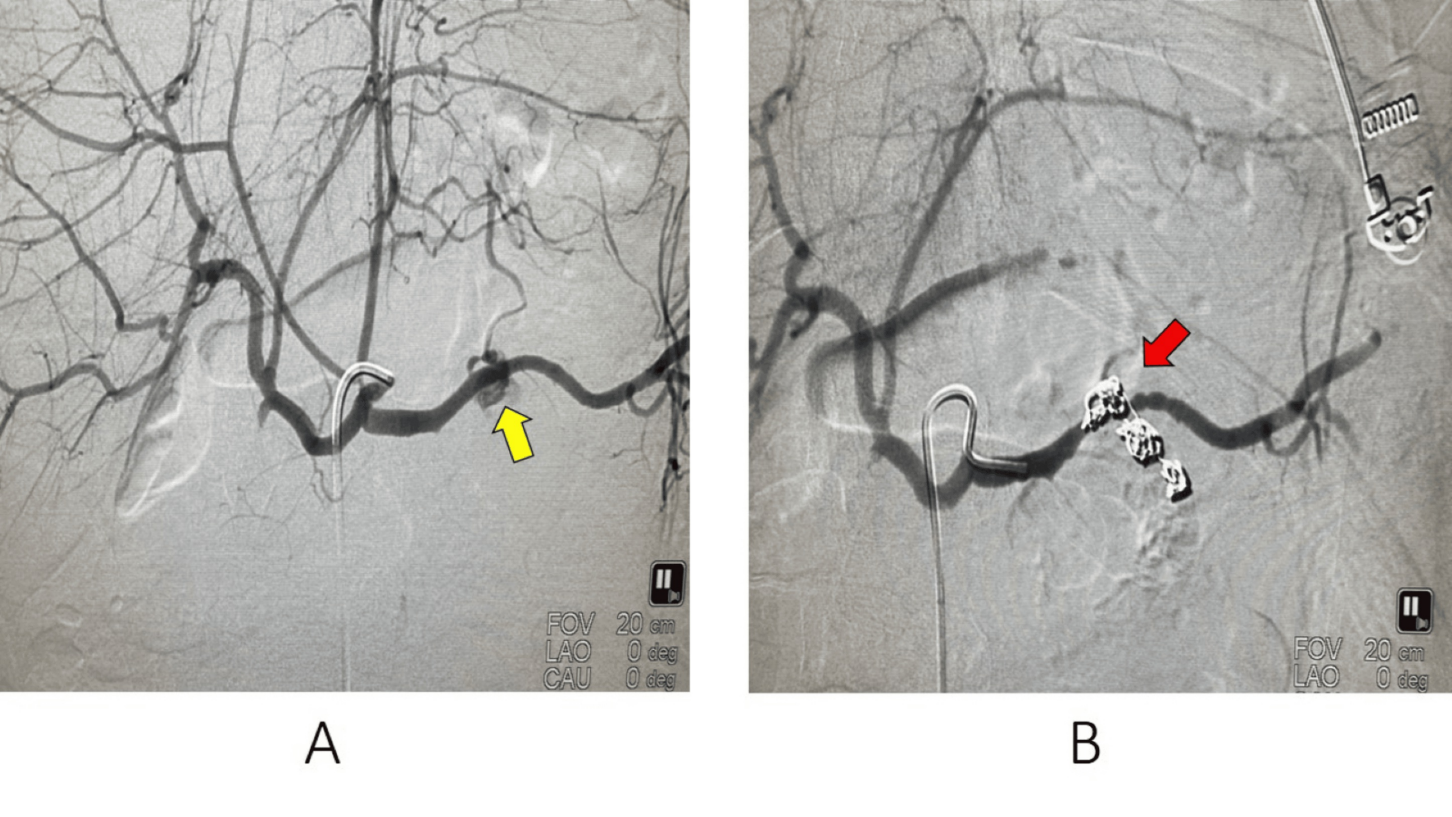
Image A: Arteriography showing the splenic pseudoaneurysm (yellow arrow); Image B: Arteriography showing complete thrombosis of the pseudoaneurysm post embolization (red arrow).

Angiography was performed under general anesthesia. The right common femoral artery was accessed, and a 5F introducer was positioned in the external iliac artery. Successive catheterization of the superior mesenteric artery, celiac trunk, common hepatic artery, and splenic artery was performed. Angiograms revealed a centimeter-sized pseudoaneurysm near the origin of a pancreatic arteriole arising from the middle third of the splenic artery, which was actively bleeding toward the pancreatic body. A 2.7F microcatheter was then advanced and placed in the pancreatic arteriole, which was embolized using 11 microcoils. These coils were placed downstream and upstream of the pseudoaneurysm until complete thrombosis was achieved. The final arteriogram showed excellent results, with no additional bleeding sites and full preservation of the main splenic artery (Figure 2).

After the procedure, the patient remained hemodynamically stable, with no further active gastrointestinal bleeding. His hemoglobin level stabilized at 10 g/dL nine days later, and he was discharged home.

## Discussion

Our case represents a rare form of acute upper gastrointestinal bleeding in a patient with recurrent acute pancreatitis complicated by pseudocyst and pancreatic ascites. A comprehensive workup and investigations were conducted to reach the correct diagnosis due to the intermittent nature of the massive bleeding associated with hemosuccus pancreaticus.

Hemosuccus pancreaticus is a rare pathology that describes bleeding from the duodenal papilla from the pancreatic duct. The most common causes of hemosuccus pancreaticus are chronic or hereditary pancreatitis. A pancreatic pseudocyst, present in around 10% to 17% of chronic pancreatitis cases, would further increase inflammation (thought to be secondary to an increase in elastase secretion) and cause lysis of the vessels’ wall, resulting in hemorrhage [3].

Upper endoscopy is the investigation of choice in cases of upper gastrointestinal bleeding. In patients with hemosuccus pancreaticus, it may reveal blood oozing from the ampulla of Vater. However, initial endoscopic evaluation is often negative for active bleeding due to the intermittent nature of the hemorrhage [2]. This was evident in our patient, who initially developed melena with a negative first gastroscopy, followed by marked active bleeding from the ampulla of Vater, as shown in the second gastroscopy.

In the literature review, we found various etiologies of hemosuccus pancreaticus. First, a case reported by Nasr D et al. presented with melena and abdominal pain, with a history of chronic pancreatitis. The patient was found to have a gastroduodenal artery pseudoaneurysm associated with a pancreatic pseudocyst, which was successfully managed with angioembolization [3]. Additionally, Leshen M et al. reported a case of hemosuccus pancreaticus in a patient with chronic pancreatitis due to a splenic artery aneurysm. The bleeding was controlled by percutaneous transsplenic embolization, as endovascular access was difficult [4]. Another case described by Harima H et al. involved melena caused by a pancreatic tumor. The patient failed coil embolization of the left gastric artery and splenic artery branches and was not a candidate for surgery due to the tumor’s invasion of surrounding organs. Radiation therapy was the treatment of choice, leading to the resolution of the bleeding [5]. Lastly, a very rare iatrogenic cause of hemosuccus pancreaticus after an endoscopic ultrasound-guided fine needle aspiration (EUS-FNA) was reported by Bokan G et al. This case involved bleeding from the ampulla of Vater following an EUS-FNA of a suspicious cystic lesion in the tail of the pancreas. The bleeding resolved spontaneously with no active blood loss. This highlights the rare complication of EUS-FNA and the importance of appropriate observation and management [6].

The preferred treatment method for hemosuccus pancreaticus is endovascular embolization to achieve hemostasis. This technique is reliable, minimally invasive, and has a success rate of approximately 79% to 100%. Coils are commonly used as embolic materials, but other materials, such as n-butyl-cyanoacrylate, Spongostan, and thrombin, can also be used. The rebleeding rate is approximately 37% in patients who undergo vascular embolization. While the specific mechanism of rebleeding is unclear, it may be related to the opening of collateral vessels [7].

If the patient is unstable with uncontrolled hemorrhage, persistent shock, and embolization is not feasible, surgical treatment is indicated. Surgical procedures may involve resection, laparoscopic techniques, or ligation of bleeding vessels. Most surgical series report a success rate of 70% to 85%, with mortality rates ranging from 20% to 25% and rebleeding rates between 0% and 5% [8]. Angiographic embolization can be a definitive treatment in the absence of pancreatitis-related indications for surgery. The treatment is traditionally surgical or by interventional radiological means [9]. If there are pancreatitis-related indications for operation, angiographic embolization may allow an elective operative procedure based on structural changes in the pancreas. If embolization fails, pancreatic resection is usually required, often on an emergent basis [10].

## Conclusions

This case highlights the rarity and complexity of hemosuccus pancreaticus as a cause of upper gastrointestinal bleeding, particularly in pediatric patients with recurrent acute pancreatitis. Early recognition and thorough investigation are essential due to the intermittent nature of the bleeding. A second-look esophagogastroduodenoscopy is crucial for identifying the source of bleeding and guiding appropriate management strategies. In this case, successful control of the bleeding was achieved through angiography and embolization, underscoring the importance of individualized, multidisciplinary treatment approaches for this rare and challenging condition.
